# Molecular landscape for risk prediction and personalized therapeutics of castration-resistant prostate cancer: at a glance

**DOI:** 10.3389/fendo.2024.1360430

**Published:** 2024-06-03

**Authors:** Jingang Jian, Xin’an Wang, Jun Zhang, Chenchao Zhou, Xiaorui Hou, Yuhua Huang, Jianquan Hou, Yuxin Lin, Xuedong Wei

**Affiliations:** ^1^ Department of Urology, The First Affiliated Hospital of Soochow University, Suzhou, Jiangsu, China; ^2^ Department of Urology, The Fourth Affiliated Hospital of Soochow University, Suzhou, China; ^3^ Department of Urology, Tongji Hospital, School of Medicine, Tongji University, Shanghai, China; ^4^ Center for Systems Biology, Department of Bioinformatics, School of Biology and Basic Medical Sciences, Soochow University, Suzhou, China

**Keywords:** castration-resistant prostate cancer, molecular signatures, carcinogenic mechanisms, personalized medicine, medical systems biology

## Abstract

Prostate cancer (PCa) is commonly occurred with high incidence in men worldwide, and many patients will be eventually suffered from the dilemma of castration-resistance with the time of disease progression. Castration-resistant PCa (CRPC) is an advanced subtype of PCa with heterogeneous carcinogenesis, resulting in poor prognosis and difficulties in therapy. Currently, disorders in androgen receptor (AR)-related signaling are widely acknowledged as the leading cause of CRPC development, and some non-AR-based strategies are also proposed for CRPC clinical analyses. The initiation of CRPC is a consequence of abnormal interaction and regulation among molecules and pathways at multi-biological levels. In this study, CRPC-associated genes, RNAs, proteins, and metabolites were manually collected and integrated by a comprehensive literature review, and they were functionally classified and compared based on the role during CRPC evolution, i.e., drivers, suppressors, and biomarkers, etc. Finally, translational perspectives for data-driven and artificial intelligence-powered CRPC systems biology analysis were discussed to highlight the significance of novel molecule-based approaches for CRPC precision medicine and holistic healthcare.

## Introduction

Prostate cancer (PCa) is the most frequently diagnosed cancer in men, representing 29% of all male cancer cases and ranking second only to lung cancer in terms of fatalities (1). The incidence and mortality of PCa in Asia are much lower than those in Europe and in the United States, but the increasing trend is much higher. The incidence of PCa is influenced by multiple factors such as age, race, and genetics, etc., and biological characteristics of the tumor, as well as the prognosis, can vary significantly among different individuals and populations. In 1941, Huggins and Hodges discovered that PCa could be treated by castration. In the early stage of the tumor, almost all PCa patients are responsive to androgen deprivation therapy (ADT). However, after a median of 18 to 24 months of treatment, nearly all patients progressed to castration-resistant prostate cancer (CRPC) (2). CRPC is a heterogeneous status with complex molecular characteristics, and its poor prognosis and high mortality rate remain to be a significant clinical challenge.

The occurrence and development of CRPC result from interactions among various carcinogenic mechanisms, which are not fully deciphered. Currently, chemotherapy, novel endocrine therapy, and immunotherapy have been used for CRPC clinical treatment, and these methods may be effective during the initial stages. However, drug resistance typically develops soon. CRPC is generally a fatal condition, with a median time to death of 1–2 years after entering this stage (3). To fight against this dilemma, biomarkers across different biological levels, e.g., genes, RNAs, proteins, and metabolites, were identified by both computational and experimental techniques for early prediction, precision prognosis and personalized therapy of CRPC, and this has increased the flourishing of molecule-based approaches for CRPC application (4).

Due to the high heterogeneity in CRPC evolution, the reliability and efficacy of current therapeutic strategies for CRPC clinical practice are still unsatisfactory. Two important issues are widely concerned across CRPC studies, i.e., what are the key signatures that could be used for indicating the development of CRPC, and what therapeutic schedules should be applied when a patient has been diagnosed with CRPC. With the accumulation of multi-omics biomedical data and technologies, a great number of biological molecules have been identified for CRPC risk prediction and personalized therapeutics.

**Figure 1:**
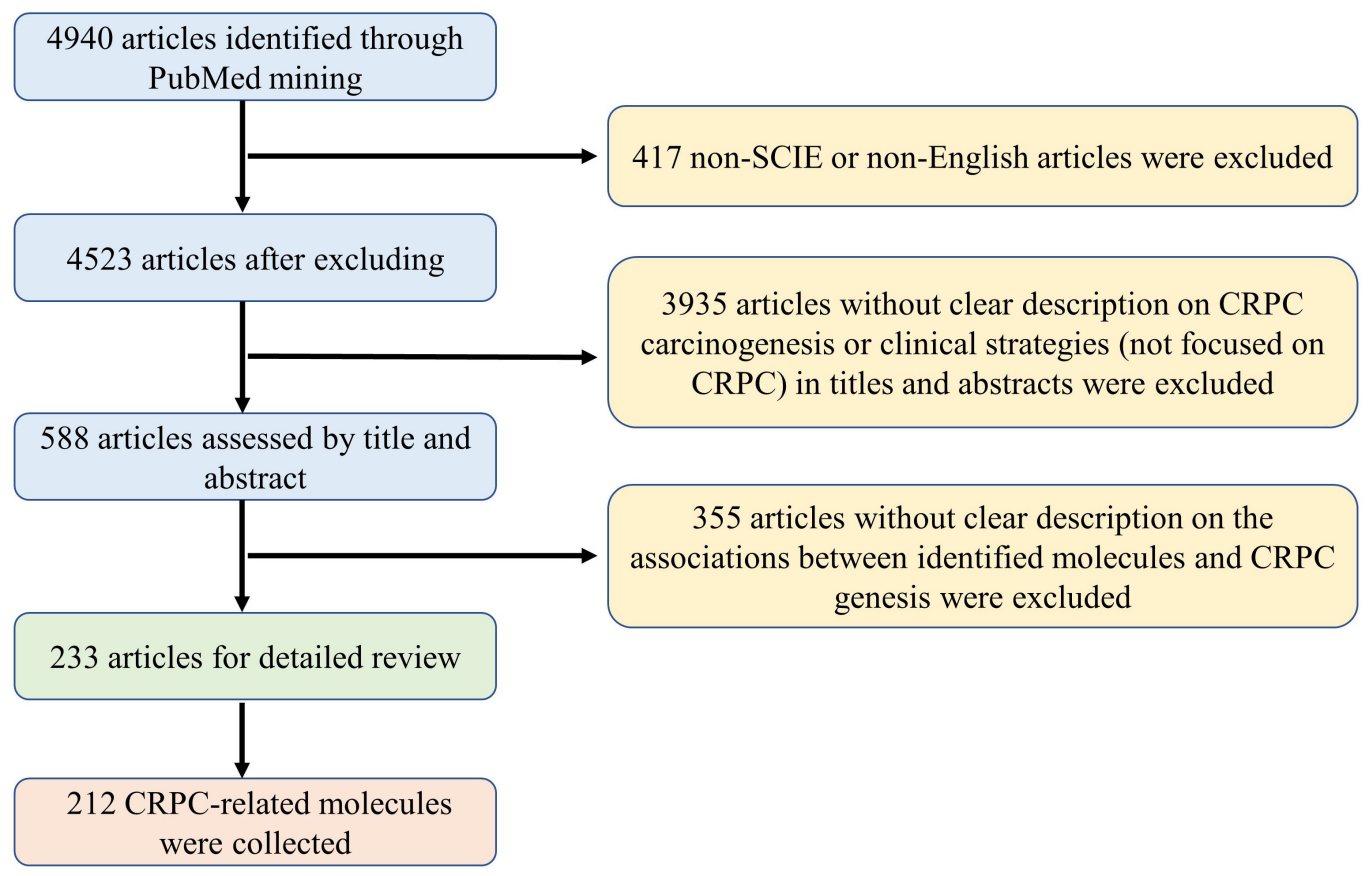
The flowchart and standards used for literature selection in this study.

In this study, a systematic literature search was conducted to collect reported CRPC-associated molecules, e.g., genes, RNAs, proteins and metabolites etc., using the NCBI PubMed up to September 2023. The search formula was defined as “prostate cancer[tiab] AND [CRPC(tiab) OR castration-resistant (tiab)] AND [gene*(tiab) OR pathway*(tiab) OR signaling*(tiab)].” As shown in **Figure 1**, a total of 4940 articles were obtained from NCBI PubMed using the above search criteria. Among them, 417 articles that were not indexed in Science Citation Index Expanded or not written in English were excluded. After reviewing the titles and abstracts, 3935 articles that were not focused on CRPC studies, i.e., unrelated to the pathogenesis or clinical prevention and therapeutic strategies of CRPC, were excluded. Based on a detailed review of the remaining 588 articles, a total of 233 articles with clear description on the associations between identified molecules and CRPC genesis were included and analyzed from three perspectives: First, introducing the carcinogenesis and clinical strategies for CRPC prevention and treatment based both on androgen receptor (AR)-related and non-AR-based mechanisms. Then, conducting a comprehensive functional characterization from single molecules to integrated pathways at three aspects, i.e, drivers promoting CRPC occurrence and progression, suppressors inhibiting CRPC development, and biomarkers indicating the state transition into CRPC. Finally, discussing future directions for CRPC precision medicine and personalized therapy to indicate novel approaches and opportunities for data-driven translational CRPC studies.

## CRPC carcinogenesis and clinical intervention strategies

### AR-related mechanisms and therapeutic schemes

**Figure 2:**
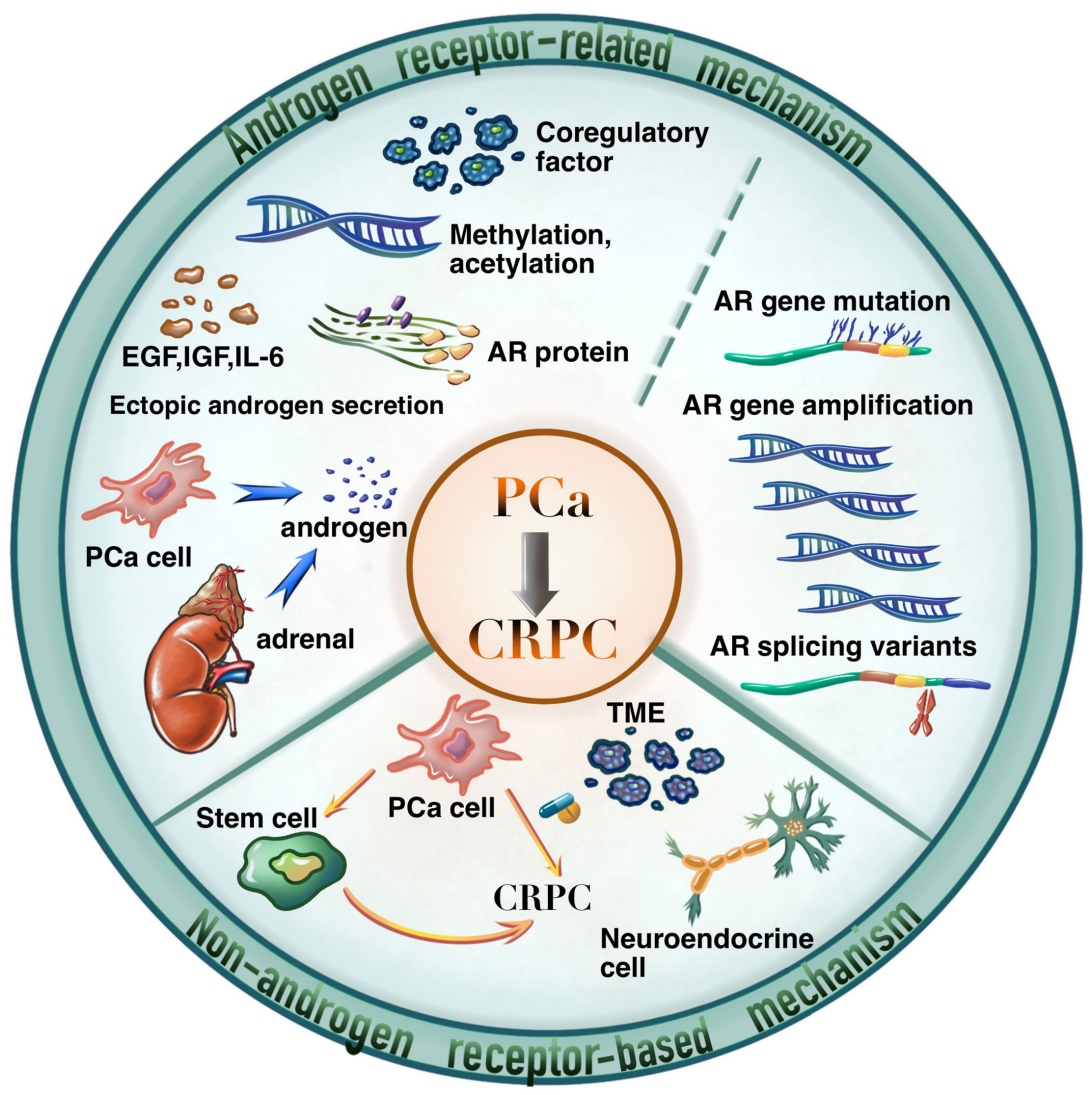
The mechanisms of PCa developed into CRPC.

As shown in **Figure 2**, the carcinogenesis of CRPC could be divided into two primary aspects, i.e., the AR-related mechanisms, and the non-AR-based mechanisms. Among them, AR-related mechanisms have been widely concerned by researchers and clinical practitioners, including AR overexpression, mutations, and splice variants, abnormal AR transcription and modifications, AR-related alternative pathway activation, and abnormal androgen synthesis (5).

**Figure 3:**
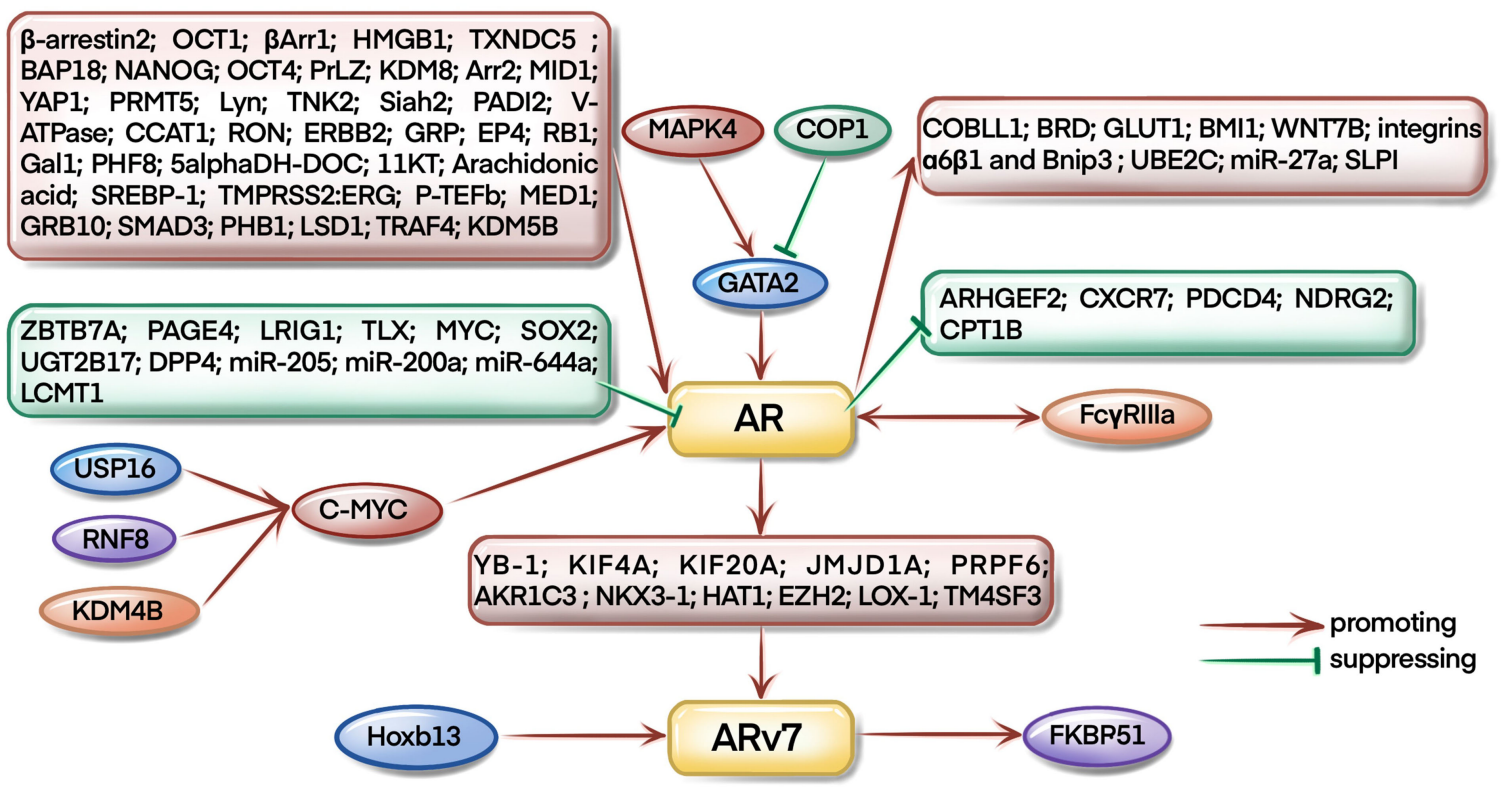
AR and ARv7-related pathways in CRPC occurrence and development.

As a famous star in CRPC development, targeting AR signaling axis has already been the first-line approach for CRPC therapy. As illustrated in **Figure 3**, most of the studies focus on the upstream regulation of AR pathway during CRPC evolution. For example, USP16, KDM4B, and RNF8 could regulate AR signaling by mediating the expression of c-myc (6–8). Interestingly, Larsson et al. found that FcγRIIIa receptors could interact with AR receptors and affect the progression of CRPC in xenograft mouse models (9). COP1 promoted GATA2 degradation to inhibit AR expression and activation (10), however, Shen et al. found that MAPK4 activated GATA2 to regulate AR transcription in mice (11).

CRPC exhibits substantial heterogeneity in terms of its sensitivity to ADT, tissue histopathological types, and genetic profiles. In patients with metastatic CRPC (mCRPC), the occurrence rate of SPOP mutations is relatively low, however, patients carrying SPOP mutations have a relatively better prognosis and are more sensitive to treatment with novel anti-androgen drugs (12, 13). In addition, Taplin et al. found that the detection rate of ARv7 in mCRPC patients was less than 10% in a randomized trial (14), but ARv7 positive patients had poor response to treatment with novel anti-androgen drugs (15). Such variances directly impacted the responsiveness (or resistance) of patients with the same histopathological type to medications. In clinical practice, corresponding theoretical support for a uniform treatment approach to CRPC is still limited, making it challenging to achieve the desired therapeutic outcomes. Deeper exploration of the heterogeneity of CRPC among patients, identifying relevant molecular targets, understanding how these targets vary among different patient subgroups or racial populations, and how this affects treatment outcomes are significant for the personalized management of CRPC patients. With the rapid advance of sequencing techniques, the next-generation sequencing (NGS) is increasingly being widely utilized in clinical diagnosis and treatment. Therefore, it has become essential to analyze the mechanisms and pathological characteristics of CRPC, to categorize CRPC patients accordingly, and to develop personalized drug dosing plans to achieve optimal treatment outcomes.

### Non-AR-based mechanisms for CRPC management

The understanding of non-AR-based mechanisms of CRPC opens new avenues for the development of novel therapies against resistance. As shown in **Figure 2**, the non-AR-based mechanisms are scattered but could be summarized as the following aspects according to recent literature reports.

Neuroendocrine cell-related mechanisms: Neuroendocrine CRPC could be induced by treatments such as ADT, radiotherapy, and chemotherapy, where neuroendocrine differentiation of PCa cells is the main driving force of disease development. Neuroendocrine CRPC exhibits resistance to hormone therapy with rapid progresses but does not reveal an elevation in PSA levels (16). Previous studies indicated that neuroendocrine cells were negative for PSA, and were more abundant in CRPC tumors (17). Moreover, neuroendocrine cells expressed IL-8, and CXCR2, and IL-8/CXCR2 had a significant role in benign and malignant neuroendocrine cells by interacting with p53 signaling (18). Li et al. demonstrated that CXCR2 expression could alter the phenotype of PCa cells, and the inhibition of CXCR2 expression in neuroendocrine PCa cells had the significance to re-sensitized enzalutamide-resistant PCa to enzalutamide (19).

Prostate stem cell-related mechanisms: It mainly includes the transformation of normal stem cells into malignant cells and the activation of tumor stem cells from differentiated tumor cells in response to external stimuli. Here a small subset of cells expressing CD44+/α2β1/CD133+ and lacking of AR expression are identified as prostate cancer stem cells (PCSC), and they hold the ability of proliferating even in androgen-depleted environments or under ADT (20). The research progress in targeted therapy for PCSC includes approaches targeting the prostate CSC microenvironment, targeted nanoparticles, and CAR-T cells targeting the CSC marker epithelial cell adhesion molecule (EpCAM), and some of them have already been entered into clinical trials (21, 22).

The molecular heterogeneity and variability of cellular populations within tumor microenvironment (TME): Chen et al. performed single-cell sequencing and discovered the activated endothelial cells, KLK3-high T-cell clusters, and KLK3-positive T cells in TME for CRPC progression to elucidate the significant variability presented in PCa and offered insights for pinpointing therapeutic targets and developing robust tumor biomarkers (23). In recent years, cancer immunotherapy has garnered increasing attention in cancer therapeutics. Some small-molecule tyrosine kinase inhibitors, whether used as single agents or in combination with other immunotherapies, may potentially improve clinical outcomes (24).

Deregulations in pathways including PI3K-Akt-mTOR, Wnt, Hippo, Hedgehog, and Notch etc: The PI3K-Akt-mTOR signaling pathway played a crucial role in regulating cell survival, proliferation, differentiation, and angiogenesis. It is recognized as one of the important pathway implicated in driving the progression of CRPC (25). In a randomized study conducted on mCRPC patients who had undergone prior docetaxel chemotherapy, the combination of the Akt inhibitor Ipatasertib with abiraterone was compared to abiraterone alone. It was observed that patients with PTEN loss derived a radiographic progression-free survival (rPFS) benefit from varying doses of Ipatasertib in conjunction with abiraterone (26). Robinson et al. identified abnormalities within the Wnt pathway in 18% of mCRPC patients. These abnormalities encompassed periodic alterations in adenomatous polyposis coli, β-catenin, and R-spondins within the pathway, implying a potential pivotal role of this pathway in CRPC progression (27). Currently, small molecule drugs and biological agents directed at the Wnt pathway remain in early stages of research, thus further exploration of the potential anti-tumor mechanisms induced by Wnt pathway inhibition needs to be conducted.

Mutations in the genetic architecture: In addition to epigenetic changes, molecular mutations in specific genes were found to be associated with the prognosis of patients with CRPC, which could guide clinical treatment for patients. In CRPC, inherited or systemic mutations, particularly alterations in the BRCA1 and BRCA2 genes, were linked to an unfavorable prognosis (28). In patients with metastatic hormone-sensitive PCa and mCRPC, TP53 mutations (32%) and PTEN mutations or copy number variations (20%), along with RB1 copy number variations (6%), were commonly observed (29). These genetic alterations were significantly correlated with increased tumor burden and a less favorable clinical prognosis (30, 31).

## Functional classification of CRPC-related signatures: from single molecules to integrated pathways

### Drivers promoting CRPC occurrence and progression

#### Genes positively associated with CRPC progression

Mutation and abnormal expression of genes enriched in AR regulation play a pivotal role in CRPC progression. As shown in **Table 1**, more than half of the retrieved genes were involved in the regulation of AR signaling. For example, YB-1, KIF4A, KIF20A and PRPF6 have been found to regulate AR and ARv7 transcription and splicing (32–35). The progression of CRPC is a process of cross-talk among multiple signaling pathways, and some genes have been shown to regulate multiple signaling pathways. For example, Choi et al. found that the knockdown of ISL1 inhibited AR signaling and AKT/NF-κB signaling and promoted enzalutamide resistance in CRPC through epithelial to mesenchymal transition (36). PROS, PKIB and PCDH7 regulated the progression of CRPC by mediating the PI3K/AKT signaling pathway (37). In addition to mRNA transcript changes, the alternations in protein abundance, e.g., TXNDC5, SREBP-1, OCT1, β-arrestin2, and p66Shc, would also contribute to the development of CRPC (38–41). It should be noticed that AR mutations are seldom occurred in the early stages of PCa, whereas aberrant AR signal transduction and alterations in AR-related pathways are prevalently observed in advanced PCa (42). Thus, early detection of AR-related molecular alterations could offer insightful opportunities for CRPC precision diagnosis and prevention.

**Table 1:** Drivers promoting CRPC occurrence and progression.

Type/name	Expression	Pathway	Function	PMID
Gene				
YB-1	↗	YB-1/AR axis	YB-1 can regulate the expression of AR and ARv7 to facilitate the advancement towards CRPC.	33064355
P-TEFb	N/A	AR	P-TEFb’s role in promoting the progression of CRPC by regulating the activity of AR protein.	28062857
HMGB1	↗	AR	HMGB1 interacts with AR to promote the advancement of CRPC.	36129149
VAV3	↗	AR	VAV3 has the potential to control the activity of the AR and drive to the progression of CRPC.	21765461
KIF4A	↗	AR/ARv7	The activation of KIF4A enhances the transcriptional activity of AR and inhibits CHIP-mediated degradation of both AR and ARv7.	31796514
TXNDC5	↗	AR	TXNDC5 enhances AR’s stability, consequently augmenting its transcriptional activity.	25500540
BAP18	↗	AR	BAP18 facilitates the progression of PCa towards castration resistance by controlling AR-induced transactivation.	27226492
NRP2	N/A	AR	NRP2 enhances the expression of genes specific to CRPC via stabilizing the complex formed between AR and nuclear pore proteins.	35754042
Hoxb13	N/A	ARv7	Hoxb13 controls the activation of ARv7, contributing to the development of CRPC.	29844167/24096478
KIF20A	↗	AR	KIF20A regulates the autocrine activation of AR and participate in the progression of CRPC.	35418689
DBC1	N/A	ARv7	DBC1 actively mediates the DNA binding and stability of ARv7, consequently advancing the progression of CRPC.	29249800
MED1	↗	AR	MED1 is excessively expressed driven by ERK and AKT signaling, which regulates the expression of AR.	23538858
GRB10	↗	AR	GRB10 is linked to the development of PCa through its interaction with PP2A.	33038264
COBLL1	↗	AR	COBLL1 promotes PCa cells growth and migration and its expression is associated with the prognosis of PCa patients.	29686105
PRPF6	↗	AR/ARv7	PRPF6 enhances AR-FL and ARv7-induced transactivation to promote the evolvement of CRPC.	33390843
ARHGEF2	N/A	AR	ARHGEF2 is important for the growth, lethal phenotype, and survival of CRPC cells and tumor xenografts.	36335093
BRD	↗	AR	BRD is highly expressed in CRPC, which is related to the transcription of AR and promotes the progression of PCa.	28591577/29555663
SREBP-1	N/A	AR	SREBP-1 induces and promotes the growth, migration, invasion, and castration-resistant progression of PCa cells *in vitro* and *in vivo*.	22064655
Arr2	N/A	AR	Arr2 can facilitate the progression of PCa by regulating the activation of AR.	25109335
GLUT1	N/A	AR	As a target of the AR, GLUT1 plays a role in the advancement of CRPC, and elevated GLUT1 indicates poor prognosis.	32428663
MYC	↗	AR/ARv7	Elevated expression of MYC can advance the progression of PCa by controlling the transcription of the AR.	35562350/30820039
GATA2	↗	AR	GATA2 is a transcription factor that facilitates the expression and activation of AR.	36251994/25489091
RNF8	↗	AR/ARv7	RNF8 up-regulates the activity of AR/ARv7, thereby promoting the progression of PCa.	35428760
OCT1	↗	AR	As an AR cofactor, OCT1 promotes PCa progression by coordinating the genome-wide AR signaling pathway.	27270436
βArr1	↗	AR	The expression of βArr1 is associated with the function of enhancing AR transcription, thereby contributing to the progression of CRPC.	33692468
BMI1	↗	AR	BMI1 is directly regulated by AR to facilitate castration-resistance in PCa.	31462713
WNT7B	↗	AR	WNT7B is associated with the growth of CRPC and osteoblastic bone metastasis of advanced PCa.	23386686
integrins α6β1 and Bnip3	↗	AR	Integrin α6β1 and Bnip3 are associated with the development of CRPC and resistance to PI3K inhibitors.	32565538
TMPRSS2:ERG	↗	AR	TMPRSS2:ERG is an AR regulatory gene that is restored in CRPC and may promote tumor progression.	19584279/31638934
UBE2C	↗	AR	UBE2C is a gene targeted specifically by the AR and is required for the growth of CRPC.	21593191/21556051
PrLZ	↗	AR	PrLZ contributes to PCa progression by directly enhancing AR transactivation at castration-resistant stage.	23104178
SOX2	↗	AR	Sox2 is a gene suppressed by AR that promotes PCa towards castration-resistance.	23326489/35067686
NANOG	N/A	the AR/FOXA1 signaling axis	NANOG facilitate PCa cells to castration resistance via regulating the AR/FOXA1 signaling axis.	27867534/21499299
OCT4	N/A	AR/FOXA1	OCT4 can facilitate PCa progression via a subtype-specific cooperative transcription factor network.	34145268
ISL1	↗	AR and EMT	ISL1 plays an important part in EMT, furthermore, it can promote the cell growth and activity of the AR.	34753990/33864110
MID1	↗	AR, Akt-, NFκB-, and Hh-pathways	Elevated MID1 expression potentially boosts the AR by amplifying Akt, NFκB, and hh signaling, thereby advancing the progression of CRPC.	24913494
PCDH7	↗	ERK/MEK and PI3K/AKT signaling pathways	Knockdown of PCDH7 decreased ERK, AKT, and RB phosphorylation and reduced colony formation, decreased cell invasion, and cell migration.	31449679
PROS	↗	PI3K/AKT/Mtor pathway	PROS promotes the development of CRPC by its apoptosis-regulating property.	27342144
PKIB	↗	PKA and Akt pathways	PKIB is highly expressed in PCa and promotes PCa cells invasion through PKA and Akt pathways.	19483721
Pik3ca	N/A	AKT-mTORC1/2	Pik3ca Mutation cooperates with Pten Loss to accelerate progression and castration-resistant growth via AKT-mTORC1/2 hyperactivation.	29581176
PTTG1	↗	EMT	The overexpression of PTTG1 could promote the resistance to ADT in CRPC via inducing EMT and increasing the cancer stem cell population.	29288516
PRKAR2B	↗	Wnt/β-catenin signaling pathway and EMT	PRKAR2B can facilitate PCa metastasis via activating Wnt/β-catenin and inducing epithelial-mesenchymal transition.	29761841
FKBP51	↗	NF-κB	FKBP51 can induce NF-κB signaling and promote the progression of CRPC.	32042745
NFKB	↗	NFκB signaling pathway	NFKB signaling is upregulated in a subset of CRPC patients and correlates with disease progression.	23093296
ZRSR2	↗	the Cyclin D1 (CCND1) pathway	ZRSR2 can promote PCa cell proliferation and the cell cycle progression. Furthermore, elevator ZRSR2 was associated with poor prognosis.	33568749
KIF15	↗	EGFR Signaling Pathway	KIF15 binds to EGFR, and prevents EGFR proteins from degradation in a Cdc42-dependent manner.	34804913
GLI3	↗	Sonic hedgehog (SHH) signaling pathway	GLI3 plays an important role for the growth and migration of androgen receptor (AR)-positive PCa cells.	34610962
MED12	↗	Wnt/β-catenin signaling	The expression of MED12 was significantly associated with high proliferative activity in PCa tissues.	24938407
SEMA3C	↗	RTK pathway	SEMA3C drives activation of multiple RTKs via Plexin B11, which promotes PCa growth and resistance to AR pathway inhibition.	29348142
WNT5A	↗	the MAPK/ERK signaling pathway	WNT5A induces CRPC via CCL2 and tumor-infiltrating macrophages.	29381686
MYCN	↗	miR-421/ATM pathway	MYCN overexpression leads to the development of poorly differentiated, invasive prostate cancer.	30657058/31260412
TCF7L1	↗	IL8/CXCR2	TCF7L1 enhances the expression of IL-8 and CXCR2 and can upregulate NED and cell motility driven via IL-8/CXCR2 signaling.	34799554
PLCϵ	↗	wnt3a/β-catenin	PLCϵ regulates AR activity and involves in drug-resistance progression in CRPC.	30684266
MYBL2	↗	Hippo-YAP pathway	The overexpression of MYBL2 promotes YAP1 transcriptional activity to promote castration-resistant progression in androgen-dependent PCa cells.	33897882
GLI2	N/A	the hedgehog signaling pathway	GLI2 is an important component of hedgehog signaling pathway, and its knockdown can inhibit CRPC development in xenograft models.	32319599
NRG1	N/A	NRG1/HER3 axis	Tumor microenvironment-derived NRG1 facilitates antiandrogen resistance in PCa.	32679108
YAP1	N/A	hippo pathway	YAP1 acts synergistically with AR to shift prostate cancer from androgen-dependent to castration-resistant growth.	28230103
β-arrestin2	N/A	β-Arrestin2/FOXO1	β-arrestin2 down-regulated FOXO1 activity to promote the development of CRPC.	25752515
FOXA1	↗	TGF-β	The downregulation of FOXA1 induces TGF-β signaling, EMT, and cell motility, which promotes growth and metastasis of CRPC.	30511964
PPFIA4	↗	PPFIA4/MTHFD2	PPFIA4 promotes CRPC progression by enhancing mitochondrial metabolism via MTHFD2.	35382861
GSE1	↗	GSE1/TACSTD2	GSE1 promotes the oncogenic and recurrent phenotypes of CRPC by targeting TACSTD2.	34439112
SLFN5	↗	mTORC1	The expression of SLFN5 was high in CRPC tumors and correlated with poor patient outcome.	33985973
CDCP1	↗	CBP/p300	CDCP1 can promote the metastatic and invasive ability of PCa cells.	35513563
p66Shc	↗	ROS	p66Shc regulates CRPC cells migration through ROS-mediated activation of migration-associated proteins.	31100478
SOCS3	↗	N/A	SOCS3 plays a crucial role in the survival machinery in PCa and is overexpressed in CRPC.	19738059
annexin A1	↗	N/A	Annexin A1 promotes the nuclear localization of the epidermal growth factor receptor in CRPC.	32858191
TFF3	↗	N/A	The over-expression of TFF3 enhances ERG-mediated cell invasion in CRPC cells.	21170267
N-cadherin	↗	N/A	N-cadherin is a major cause of both PCa metastasis and castration resistance.	21057494
HIF1A	N/A	N/A	Inhibition of HIF1A induces apoptosis signaling in CRPC cells and is sensitive to androgen deprivation.	37070472
SMAD3	N/A	AR	SMAD3 facilitates expression and activity of the AR.	36727462
PHB1	↗	AR	PHB1 promotes PCa cells proliferation and invasion, and its expression is positively correlated with the prognosis of CRPC patients.	37210546
SRC-3	↗	N/A	SRC-3 is required to drive CRPC progression.	23650284
UHRF1	N/A	PI3K/AKT	AKT1 regulates the stability of UHRF1 protein, and its overexpression indicates poor prognosis.	36593255
TM4SF3	↗	AR/ARv7	TM4SF3 interacts with AR and ARv7 and promotes the recruitment of related target genes.	36951301
RNA				
HOXD-AS1	↗	H3K4me3/WDR5	HOXD-AS1 facilitate PCa progression and chemo-resistance by recruiting WDR5.	28487115
HOTAIR	↗	N/A	HOTAIR promotes neuroendocrine differentiation in CRPC.	29944905
CCAT1	↗	AR	CCAT1 is significantly upregulated in CRPC and elevated CCAT1 expression is associated with a poor prognosis.	31387890
SOCS2-AS1	↗	N/A	SOCS2-AS1 can promote the growth of castration-resistant and androgen-dependent cells and inhibit apoptosis in PCa.	27342777
SNHG17	↗	miR-144/CD51 axis	SNHG17 promotes CRPC cells proliferation and invasion via regulating the miR-144/CD51 axis.	32351538
Linc00963	↗	miR-655	Linc00963 promotes TRIM24 expression in CRPC cells by inhibiting miR-655 expression, which promotes its cell proliferation and colony-forming ability.	33643926
miR-221	↗	EMT/AR	MiR-221 downregulates HECTD2 and RAB1A to promote the progression of androgen independence in PCa cells.	23770851
miR-302/367	↗	miR-302/367/LATS2/YAP pathway	MiR-302/367 cluster can promote the progression of CRPC by down-regulating LATS2, reducing the phosphorylation of YAP oncoprotein and enhancing its nuclear translocation.	28745315
Enzymes				
JMJD1A	N/A	AR/ARv7	JMJD1A is a key co-activator of AR and is involved in the alternative splicing of ARv7.	29712835
LSD1	N/A	FOXA1/MYC-AR	LSD1 can control FOXA1 methylation and MYC signaling to adjust AR expression.	36877164
KDM3B	↗	N/A	KDM3B has a strong anti-proliferation ability and plays an important role in regulating the progression of CRPC.	31822799
KDM4B	↗	AR/c-Myc	KDM4B collaborates with c-Myc to enhance AR transcription, leading to the promotion of CRPC progression.	34335964/32617978
KDM5B	N/A	AR	An AR coregulator promotes the growth and invasive capacity of PCa.	37152294
KDM5C	↗	BRD4/KDM5C/PTEN pathway	KDM5C is transcriptionally regulated by BRD4 and promotes CRPC cell proliferation by repressing PTEN.	30921702
KDM8	↗	AR/JMJD5	KDM8 is a PCa metabolism gene regulator and androgen response gene, which can dual activate AR and JMJD5.	30072740
HAT1	↗	HAT1/AR	High expression of HAT1 can increase AR expression and is associated with resistance of CRPC cells to enzalutamide.	34323404
INMT	↗	SMYD3/INMT	The expression of INMT is significantly increased in CRPC, and elevated INMT is associated with poor clinical prognosis.	34587977
PRMT5	N/A	AR	PRMT5 cooperates with a methylosome subunit pICln, which functions as an epigenetic activator of AR transcription in CRPC.	32999000
EZH2	↗	AR/AR-Vs	EZH2 modulates oncogene activity, which promotes CRPC progression.	36300627/23239736
PKA	N/A	AR	PKA plays an important part in the nuclear translocation of AR.	30992362
Lyn	↗	AR	Lyn tyrosine kinase can regulate the stability and transcriptional activity of AR in CRPC.	25133482
TNK2	N/A	AR	TNK2 regulates the AR gene expression via adjust histone H4 Tyr88.	28609657
MAPK4	↗	AKT,GATA2/AR	MAPK4 facilitates PCa propagation and castration resistance through activating GATA2/AR and AKT signal pathway.	33586682/21559022
Etk	↗	AR	Etk can be involved in regulating AR activity to facilitate the castration-resistant growth of PCa during androgen depletion.	20570899
PKC	N/A	NF-κB pathway	PKC plays a significant role in castration resistance by controlling Twist1 expression via NF-κB in PCa.	28223364
LIMK2	↗	LIMK2/TWIST1/PTEN/SPOP	LIMK2 promotes the progression of CRPC through a variety of signaling pathways.	30716360/33311589/32931887
IKKα	N/A	IKKα/E2F1/BMI1 pathway	IKKα regulates the regeneration and tumor recurrence of PCa by modulating IKKα-E2F1-BMI1 pathway.	23796898
GCN2	N/A	N/A	GCN2 can facilitate PCa progression via maintaining amino acid homeostasis.	36107759
STYK1	↗	N/A	STYK1 is overexpressed in CRPC, and STYK1 knockdown can inhibit the growth of tumor cells, which may be a molecular target of CRPC.	19664042
mTOR	↗	N/A	Nuclear mTOR acts as a transcriptional integrator of the androgen signaling pathway in PCa.	28724614
NEK6	↗	N/A	The overexpression of NEK6 stimulated cytoskeletal, differentiation, and immune signaling pathways and maintained gene expression patterns.	27899381
Skp2	↗	N/A	Skp2 can facilitate CRPC progression and stem cell features via stabling Twist protein expression.	28346424
GPX2	↗	N/A	The overexpression of GPX2 is involved in cell proliferation and prognosis in CRPC.	24562575
AKR1C3	↗	ARv7	AKR1C3 plays an important role in steroidogenesis and facilitates the stability of ARv7.	31308078/31052459/36901944
HO-1	↗	ROS	HO-1 can reduce cell apoptosis and promote the progression of PCa cells to castration resistance.	36265795
SQLE	↗	PI3K/Akt/GSK3β pathway	SQLE increases cholesterol biosynthesis to facilitate the growth and survival of PCa cells.	35767703
Siah2	↗	AR	Siah2 regulates AR transcriptional activity to contribute to CRPC.	23518348
USP16	↗	USP16/c-Myc	USP16 can regulate the proliferation of CRPC cells through deubiquitinating and stabilizing c-Myc.	33546726
UGT2B17	↗	AR, c-Src	UGT2B17 can catabolize AR agonists into inactive forms to maintain androgen homeostasis.	27659047
PADI2	↗	AR	PADI2 can activate AR signaling by mediating citrullination in the nucleus to promote the progression of PCa.	28819028
V-ATPase	N/A	AR	V-ATPase can affect the progression of CRPC via regulating the expression of AR and AR target genes.	33563753
ACSL3	↗	intratumoral steroidogenesis	ACSL3 contributes to the growth of CRPC through intratumoral steroidogenesis.	28771887
CAPN2	↗	AKT/mTOR	CAPN2 regulates the activation of MMP-2 and MMP-9, as well as the expression of phosphorylated proteins AKT and mTOR.	28280729
CTSK	↗	IL-17/CTSK/EMT axis	CTSK promotes the tumor growth and metastasis by IL-17/CTSK/EMT axis and mediates M2 macrophage polarization in CRPC.	36138018
3βHSD1	↗	intratumoral androgen synthesis	3βHSD1 is the rate-limiting enzyme for potent androgen synthesis from extragonadal precursors.	37009898/36647826
TRAF4	↗	AR	TRAF4 can modulate the nonproteolytic ubiquitination of AR.	37155905
NEK6	N/A	redox balance	NEK6 is a central kinase in CRPC progression, and it can regulate redox balance and the DNA damage response.	36672191
KTM5A	N/A	N/A	A PCa oncogene that can regulate CDC20 through multiple pathways.	37509260
ACACA	↗	*de novo* fatty acid synthesis and PI3K/AKT signaling	ACC1 can impact CRPC by controlling *de novo* fatty acid synthesis and mitochondrial β-oxidation.	36410440
Receptor				
AR	↗	AR	Aberrant AR expression cross-talks with other oncogenic pathways, generally promoting the progression of CRPC.	24948871
RON	↗	AR	RON overexpression can activate multiple transcription factors, promoting the activation of AR response genes by AR and their nuclear localization.	30121008
TLX	↗	AR	TLX plays an oncogenic role in PCa by suppressing oncogene-induced senescence, and it can confer resistance to androgen deprivation and anti-androgens.	29555975
FcγRIIIa	↗	AR and PIP5K1α pathways	FcγRIIIa facilitates the growth and metastasis of PCa by regulating the AR and PIP5K1α pathways.	34932854
ERBB2	N/A	RTKs/AR	ErbB2 can stabilize AR protein, and the expression of ERBB2 is increased in some abiraterone-resistant PCa patients.	26936914
GRP/GRP-R	N/A	NF-κB/AR-Vs	GRP/GRP-R facilitates the CRPC progression via enhancing the expression of AR splice variants.	34461557
ARv7	↗	ARv7	ARv7 is considered a key driver of ENZR in CRPC.	30334814/30453546
EP4	↗	ARv7	Ectopic overexpression of EP4 drives PCa cells proliferation and PSA production.	20145136
LRH-1	↗	intratumoral androgen biosynthesis	LRH-1 promotes intratumoral androgen biosynthesis to facilitate castration-resistant growth of PCa.	29438990
ERRα	↗	intratumoral androgen biosynthesis	ERRα regulates intratumoral androgen biosynthesis to facilitate CRPC progression.	32226548
EGFR	N/A	EGFR-LIFR	Interplay of EGFR and signal transducer and STAT3 can mediate the progression of PCa.	32963351
AVPR1A	N/A	cAMP/protein kinase A signaling pathway	Coexpression with AVPR2 is highly associated with PCa development.	35503085
CXCR7	↗	MIF/CXCR7/AKT Signaling Pathway	The expression of CXCR7 was elevated after ADT, and it could facilitate the growth and metastasis of CRPC.	30224544/30952632
CHRM1/3	↗	FAK/YAP signaling axis	PCa patients with high expression of CHRM1 and CHRM3 are more likely to progress to CRPC.	32205868
SR-B1	↗	N/A	SR-B1 decrease steroid synthesis and steroid-independent mechanisms to impede PCa proliferation.	34575583
C5AR	↗	N/A	C5AR can promote the proliferation, invasion and PD-L1 expression of PCa cells.	33368414
FGFR1	↗	N/A	FGFR1 can drive the metastatic progression of PCa.	21952621
Notch1	↗	N/A	Notch1 promotes the progression of CRPC, and its loss will inhibit the growth and metastasis of CRPC.	31028097/27694579
LOX-1	↗	AR/ARv7	LOX1 facilitates ROS generation and NF-κB activation, and increased the expression of AR and ARv7 in CRPC.	36982155
CHRM3	↗	CaM/CaMKK pathway	The autocrine activation of CHRM3 facilitates PCa growth and castration resistance through CaM/CaMKK-mediated phosphorylation of Akt.	26071486
Others				
erythropoietin	↗	N/A	The erythropoietin was upregulation in CRPC, and it can facilitate PCa cells proliferation and invasion.	31417010
angiogenin	↗	N/A	Angiogenin facilitates the growth of androgen-stimulated PCa and actives castration resistance.	23851444
endogenous estrogen	↗	Erα/MMP12	Aromatase-induced endogenous estrogen enhances MMP12 expression via Erα, which promotes to the progression and tumor metastasis in CRPC.	31499120
DHT	N/A	GR pathway	STAT5 can be activated by DHT via GR pathway, which enhances CRPC cell proliferation.	25043756
5alphaDH-DOC	N/A	AR	5alphaDH-DOC within CRPC tissues might activate the AR pathway for proliferation and survival of CRPC cells under an extremely low level of DHT.	20560974
11KT	N/A	AR	11KT is a potent AR agonist and is the major active androgen in PCa patients after castration.	33974560
IL-6	↗	JAK/Stat3 signaling pathway	IL-6 promotes the progression from PCa to castration-resistance through multiple signaling pathways.	28865178/23536722
IL-23	↗	N/A	In androgen-deprived conditions, IL-23 promotes PCa cells proliferation via activating AR pathway signaling.	29950727
IL-4	N/A	CBP/p300/AR	IL-4 regulates AR through CBP/p300, thereby promoting the progression of PCa to a castration-resistant state.	18819102
lactate	N/A	N/A	lactate regulates the metabolic-epigenetic axis to foster metastatic potential in PCa.	35135811
arachidonic acid	N/A	AR	Arachidonic acid can induce steroidogenesis, which promotes CRPC progression via activating AR.	19790237
CD4^low^HLA-G+ T cells	↗	N/A	CD4^low^HLA-G+ T cells may drive androgen-independent PCa progression via mediating the migration and activity of CD11blowF4/80hi macrophages.	30297869
adipocyte	N/A	IL-6/leptin/JAK/Stat3 signaling axis	Adipocytes regulate PD-L1/NKG2D ligand levels in PCa cells to develop the resistance to cytotoxic action of NK cells.	29330929
Osteoclasts	N/A	N/A	Osteoclasts can directly affect the gene expression of CRPC and reduce cell apoptosis.	35971022
Platelets	N/A	N/A	Platelets can synthesize testosterone in a novel mechanism, and might act to sustain CRPC state.	26152357

N/A, not applicable; ↗, high expression.

#### RNAs involved in promoting the development of CRPC

As shown in **Table 1**, numerous studies demonstrated the role of RNAs in CRPC development. For instance, certain specific RNA molecules could modulate the proliferation and invasive capabilities of CRPC cells, consequently influencing tumor progression. In addition, RNA could serve as a molecular marker to predict the occurrence and prognosis of CRPC. In particular, several studies showed that the elevated expression of long non-coding RNAs (lncRNAs) in CRPC was related to the degree of malignancy and drug resistance of tumors. For example, HOXD-AS1 facilitated PCa progression and chemo-resistance by recruiting WDR5 (43). HOTAIR promoted neuroendocrine differentiation in CRPC (44). CCAT1 was an oncogenic factor for CRPC progression and was highly up-regulated in CRPC, and elevated CCAT1 was associated with poor prognosis (45). SOCS2-AS1 promoted the growth of castration-resistant and androgen-dependent cells and inhibited apoptosis in PCa (46). In addition, microRNAs (miRNAs) also play an important roles in CRPC, such as miR-221 and miR-302/367, and they promoted the development of CRPC by inhibiting the expression of targeted anti-tumor proteins (47, 48).

#### Enzymes that regulate CRPC progression

**Figure 4:**
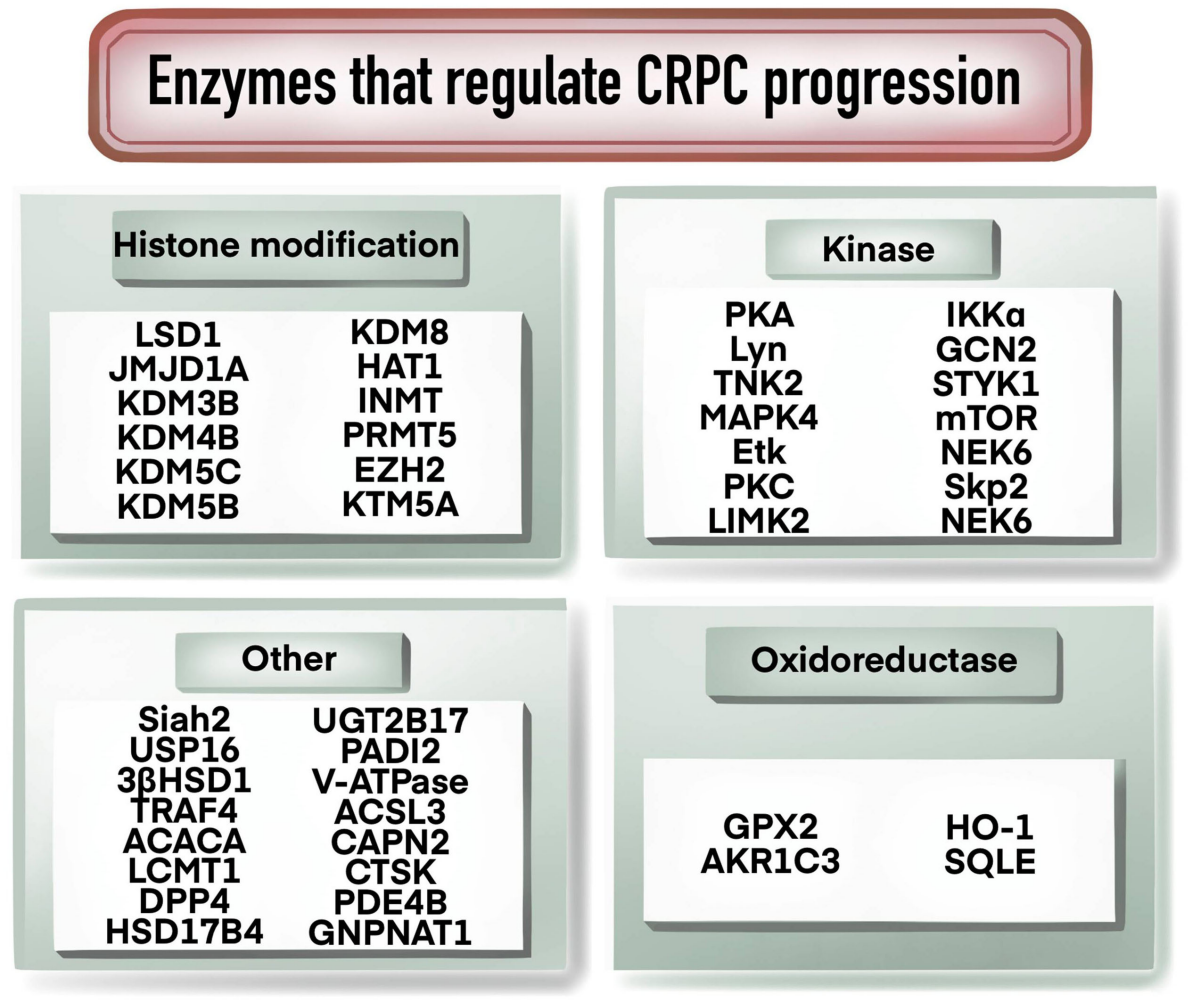
Enzymes regulating CRPC progression.

As shown in **Figure 4**, enzymes play an important role in the progression of CRPC, and many studies have been conducted on enzymes related to histone modification. For example, histone demethylases LSD1, JMJD1A, KDM3B, KDM4B, KDM5B and KDM5C were found to be involved in the regulation of AR, c-Myc or PTEN signaling pathways to affect the development of CRPC (49–54). KDM8 could double activate AR and JMJD5, participating in the regulation of androgen response and the regulation of PCa metabolism genes (55). Over-expression of HAT1 increased AR expression and was associated with the resistance of CRPC cells to enzalutamide (56). Methylation-modifying enzymes, kinases and oxidorereductases also played significant functions in CRPC development and progression. For example, INMT promoted the production or release of methylation of anticancer metabolites, and PRMT5 and EZH2 regulated the transcription of AR through methylation (57–59). Kinases such as PKA, Lyn, TNK2, MAPK4 and Etk are involved in CRPC progression by regulating AR signaling pathway (11, 60–62). PKC, LIMK2, IKKα, and GCN2 have been reported to be involved in CRPC regulation through various mechanisms, as listed in **Table 1**. Oxidoreductase such as GPX2, AKR1C3, HO-1 and SQLE have been reported to be elevated in CRPC and to contribute to CRPC progression and prognosis (63–65). Ubiquitination is a regulator in CRPC progression. For example, Siah2 regulated the transcriptional activity of AR, and USP16 promoted CRPC proliferation through deubiquitination and stabilization of c-Myc (6, 66). Other enzymes such as UGT2B17, PADI2 and V-ATPase could facilitate CRPC progression by regulating AR signaling (67–69). ACSL3 contributed to the growth of CRPC through intratumoral steroidogenesis (70). CTSK promoted the tumor growth and metastasis by IL-17/CTSK/EMT axis and mediates M2 macrophage polarization in CRPC (71).

#### Receptor molecules involved in CRPC progression

AR is reported to be functional in CRPC progression by mediating the effects of androgens. As shown in **Table 1**, it is widely acknowledged that resistance to ADT is often a result from aberrations within the AR signaling, such as mutations in AR gene or heightened expression of the AR protein. Accumulating evidence confirmed that the aberrant cross-talk between AR expression and other oncogenic pathways could promote CRPC progression (72). Some receptor molecules regulate the progression of CRPC by regulating AR directly or indirectly. For example, the overexpression of RON could activate multiple transcription factors, and it promoted AR activation of AR response genes and nuclear localization (73). FcγRIIIa facilitated the growth and metastasis of PCa by regulating the AR and PIP5K1α pathways (9). TLX plays an oncogenic role in prostate carcinogenesis by suppressing oncogene-induced senescence, and it could confer resistance to androgen deprivation and anti-androgen (74). ErbB2 stabilized AR protein, and the expression of ERBB2 was increased in some abiraterone-resistant PCa patients (75). ARv7 is considered as a key driver of ENZR in CRPC (76). Ectopic overexpression of EP4 drived PCa cells proliferation and PSA production via regulating ARv7 signaling pathway (77). Ubiquitination is an intracellular protein regulatory mechanism, which is closely related to the occurrence and progression of CRPC. It has been reported that the high expression of the ubiquitination modifying enzyme Siah2 could promote the transcriptional activity of AR and deubiquitinate the enzyme USP16 could regulate the proliferation of CRPC cells through deubiquitinating and stabilizing c-Myc. Other receptors that have been implicated in CRPC include cell surface molecular receptor and tumor immunotherapy receptor. LRH-1 and ERRα facilitate CRPC progression via promoting intratumoral androgen biosynthesis (78, 79). Interplay among EGFR and signal transducer and STAT3 could mediate the progression of PCa (80). Co-expression of AVPR1A with AVPR2 was highly correlated with the development of PCa (81). The expression of CXCR7 was elevated after ADT, and it could facilitate the growth and metastasis of CRPC via MIF/CXCR7/AKT signaling pathway (82). PCa patients with high expression of CHRM1 and CHRM3 were more likely to progress to CRPC (83). The expression of FGFR1 and Notch1 were all elevated in CRPC and they regulated the proliferation and progression of CRPC through different mechanisms (84, 85).

#### Other molecules

The transition from HSPC to the castration-resistant stage is also encompassed by hormones, cytokines, and cellular components. As described in **Table 1**, androgens are signaling molecules that are necessary for the growth and maintenance of PCa cell survival. 5alphaDH-DOC within CRPC tissues might activate the AR pathway for proliferation and survival of CRPC cells under an extremely low level of DHT (86). 11KT is a potent AR agonist and is the major active androgen in PCa patients after castration (87). Other hormones are also functional in PCa progression, tumor growth, and invasion (88–90). Cytokines are a class of secreted proteins or molecules that can regulate and influence cell-to-cell interactions and communication. In the context of CRPC, cytokines and factors mediating the interaction between tumor cells and immune cells to promote the proliferation, invasion, and metastasis of PCa cells. As stated in **Table 1**, IL-6 promoted the progression from PCa to castration resistance through multiple signaling pathways (91). In androgen-deprived conditions, IL-23 promoted PCa cell proliferation by activating the AR pathway signaling (92). Recent studies suggested a close relationship between abnormal fatty acid metabolism and CRPC progression. Lactate regulated the metabolic-epigenetic axis to foster metastatic potential in PCa (93). Some cells have also been reported to be functional in the advancement of CRPC. CD4^low^HLA-G^+^ T cells may drive androgen-independent PCa progression by mediating the migration and activity of CD11blowF4/80hi macrophages (94). Platelets could synthesize testosterone in a novel mechanism, and might sustain CRPC state (95).

### Suppressors inhibiting CRPC evolution

#### Genes that inhibit the growth of CRPC

As illustrated in **Table 2**, several genes were found to be negatively associated with CRPC progression. For example, the expression of RB1 was negatively correlated with the prognosis of CRPC patients (96). In addition, the knockdown of PLZF promoted the CRPC phenotype and facilitated the proliferation of CRPC cells in a xenograft model (97). On the other hand, several genes, i.e., PTEN, LRIG1, PAGE4, NKX3–1, ZBTB7A, and PDCD4, could regulate the AR signaling pathway through various ways to inhibit the progression of CRPC (98–103). Furthermore, DAB2IP knockdown cells showed drug resistance, and increasing DAB2IP enhanced drug sensitivity. Besides, a study also found that it could regulate the Wnt/β-catenin and IGF-I signaling pathways (104). KLF5 downregulation increased the expression of BECN1 and induced cell autophagy in PCa. It could also desensitize CRPC cells to docetaxel through the AMPK/mTOR/p70S6K signaling pathway (105).

**Table 2:** Suppressors inhibiting CRPC evolution.

Type/name	Expression	Pathway	Function	PMID
Gene				
PTEN	↘	N/A	PTEN loss and β-Catenin activation synergistically promote AR-independent CRPC progression.	31719098/26379078
LRIG1	↘	AR	LRIG1 is pleiotropic AR regulated tumor suppressor, elevator LRIG1 hint a better prognosis of PCa patients.	31792211
FOXO1	↘	PTEN/AR	FOXO1 binds to the TAU5 motif in the AR NTD and inhibits ligand-independent activation of AR splice variants.	23389878
PAGE4	↘	AR	PAGE4 promotes progression to advanced lethal PCa via regulating AR signaling.	22885105
NKX3–1	↘	AR, ARv7, and AKT signaling	Downregulation of NKX3–1 is the mechanism driving the pathogenesis of CRPC.	34625072/34066036
ZBTB7A	↘	AR	ZBTB7A can inhibit the growth and recurrence of CRPC by mediating the transcriptional repression activity of the AR.	31444154
PDCD4	N/A	AR/PDCD4	PDCD4 is an androgen-suppressed protein that can regulate PCa cell proliferation, apoptosis, and castration resistance.	30518628
TCF7	N/A	AR/miR-1/TCF7	TCF7 is inhibited by AR through miR-1-mediated downregulation and participates in the progression of resistance to ADT in PCa.	28220803
NDRG2	N/A	AR, N-myc/NDRG2	The expression of NDRG2 is negatively correlated with that of AR and c-Myc.	25756511
DAB2IP	↘	Wnt/β-catenin and IGF-I signaling	DAB2IP knockdown cells show drug resistance, and increasing DAB2IP will enhance drug sensitivity.	23838317/26512963
KLF5	↘	AMPK/mTOR/p70S6K signaling pathway	KLF5 downregulation can increase the expression of BECN1 and induce cell autophagy in PCa and can desensitize CRPC cells to docetaxel.	31534497
RB1	↘	N/A	RB1 is often absent in CRPC and is associated with a poor prognosis.	34975152/36928314
PLZF	N/A	N/A	The knockdown of PLZF promotes the CRPC phenotype and facilitates the proliferation of CRPC cells in a xenograft model.	25808865
RNA				
miR-27a	↘	AR, PI3K/Akt	MiR-27a can be down-regulated by AR and PI3K, which promotes CRPC progression.	27594411
miR-205	↘	AR	The expression of miR-205 is low in CRPC, and it can negatively regulate AR.	23571738
miR-200a	↘	BRD4/AR	MiR-200a can inhibit the BRD4/AR signaling pathway, and its high expression indicates good prognosis.	30784214
miR-146a	↘	MiR-146a/EGFR	MiR-146a inhibits the expression of MMP2, which blocks the growth and development of PCa cells.	22161865
miR-145–5p	↘	SOX11/MYCN axis	MiR-145–5p suppress neuroendocrine differentiation and tumor progression via SOX11/MYCN axis.	35368699
miR-452	↘	miR-452/WWP1	MiR-452 suppresses PCa cells migration and invasion by modulating WWP1.	27070713
DRAIC	↘	NF-κB signaling pathway	DRAIC inhibits the growth of PCa by suppressing NF-κB activation via interacting with IκB kinase.	31900260
miR-200b-3p/200c-3p	↘	PRKAR2B/Wnt/β-catenin signaling	MiR-200b-3p/200c-3p facilitates the PCa progression by mediating transcriptional regulation of PRKAR2B.	31986411
miR-644a	↘	EMT/Warburg Effect	MiR-644a mediates tumorigenesis in CRPC patients via disrupting the Warburg effect.	30808676
Enzymes				
LCMT1	N/A	AR	A suppressor of AR addicted PCa, which can inhibit tumor growth.	37644036
DPP4	↘	AR	Depletion of DPP4 enhances growth factor activity, and inhibition of DPP4 accelerates the emergence of PCa resistance.	30242112
PDE4B	↘	PDE4B/PKA pathway	PKA pathway can be activated by downregulation of PDE4B, which contributes to the progression of PCa.	22529021
GNPNAT1	↘	PI3K/AKT signaling pathway	CRPC-like cells with loss of GNPNAT1 function exhibited enhanced proliferation and invasion.	27194471
HSD17B4	↘	N/A	Loss of HSD17B4 blocks androgen inactivation and promotes CRPC progression.	29346776

N/A, not applicable; ↘, low expression.

#### RNAs involved in inhibiting CRPC progression

MicroRNAs (miRNAs) are small non-coding RNA molecules that can regulate gene expression post-transcriptionally by binding to target mRNAs and inhibiting their translation or promoting their degradation. As shown in **Table 2**, many studies have focused on the correlation between miRNA and PCa progression. The AR signaling pathway represents the classical route of progression in CRPC. In this study, miR-205, and miR-200a were found to regulate AR signaling through different pathways to inhibit the progression of CRPC. Among them, the high expression of miR-200a indicated good prognosis (106, 107). In addition, miR-452 suppressed PCa cells migration and invasion by modulating WWP1 (108). MiR-200b-3p/200c-3p inhibited the PCa progression by mediating transcriptional regulation of PRKAR2B (109). MiR-644a mediated tumorigenesis in CRPC patients via disrupting the Warburg effect (110). In addition to miRNAs, lncRNAs are also a class of non-coding RNA molecules in CRPC development. For example, DRAIC could inhibit the growth of PCa by suppressing NF-κB activation via interacting with IκB kinase (111).

#### Enzymes that inhibit the development and progression of CRPC

As shown in **Table 2**, there is a paucity of studies investigating the inhibitory effects of enzymes on CRPC occurrence and progression. Rasool et al. discovered and demonstrated in murine models that the loss of heterologous LCMT1, along with biased protein phosphatase 2A activity, drived the progression of PCa and confers resistance to treatment (112). Depletion of DPP4 enhanced growth factor activity, and inhibition of DPP4 accelerated the emergence of PCa resistance. Kashiwagi et al. discovered that depletion of DPP4 augments growth factor activity, while inhibition of DPP4 expedited the emergence of PCa resistance (113). The study conducted by Ko et al. revealed that the emergence of CRPC was facilitated by the loss of a specific splice form of HSD17B4, which was responsible for inactivating androgen hormones (114). In addition, CRPC-like cells with loss of GNPNAT1 function exhibited augmented proliferation and invasion (115).

### Biomarkers indicating the state transition into CRPC

Biomarkers are important predictors indicating the state change for CRPC management. Previous studies identified plenty of potential biomarkers that may help the early detection, prognosis, and treatment response prediction of CRPC patients. As listed in **Table 3**, HSD3B1 is a biomarker that enhanced dihydrotestosterone synthesis from extra-gonadal precursors and has been shown to predict castration resistance in PCa in two retrospective studies (116, 117). PHF8 could promote CRPC progression through the HIF/PHF8/AR axis (118). The expression of Gal-1 in CRPC cells was significantly higher than that in hormone-sensitive PCa cells (119). MiR-32, FOXC2, and miRNA-221/222 have been found to be potential biomarkers for the progression and malignant invasion of CRPC (120–123). LIF was associated with CRPC neuroendocrine and could be used as a serum biomarker for the diagnosis of advanced PCa (124).

**Table 3:** Biomarkers indicating the state transition into CRPC.

Type/name	Expression	Pathway	Function	PMID
MED15	↗	TGF-β/AR	MED15 is overexpressed in CRPC and mCRPC patients, but it is low or no expressed in hormone sensitive PCa and benign prostate tissue.	24374838
stanniocalcin 2	↗	N/A	Stanniocalcin 2 overexpression in CRPC and aggressive PCa, and it no expressing in benign prostate tissue.	19298603
SLPI	↗	AR	SLPI is required for CRPC cell proliferation under androgen deprivation conditions, and increasing obviously in mCRPC.	26876202
sdRNAs	↗	N/A	sdRNAs promotes chemotherapeutic resistance, anc can be a new biomarker for CRPC clinical intervention.	35455981
SOX7/9	↗	N/A	During castration resistance, SOX9 was obviously increased and SOX7 was obviously increased.	22703285
Gal 1	↗	AR	The expression of Gal-1 in CRPC cells was significantly higher than that in hormone sensitive PCa cells.	29666302
HSD3B1	N/A	N/A	HSD3B1 is a predictive biomarker of CRPC patients.	29049452/27575027
PHF8	↗	HIF/PHF8/AR axis	PHF8 can regulate the PCa progression via AR signaling pathway.	27991916
miR-32	↗	N/A	The overexpression of miR-32 results in reduced expression of BTG2 in CRPC, making it a potential marker for aggressive disease.	22266859/35228520
NR6A1	↗	N/A	Cellular levels of NR6A1 are associated with the progression of PCa and can serve as a biomarker for PCa invasiveness.	23532770
LIF	↗	ZBTB46/LIF axis	LIF can activate ZBTB46 to promote CRPC and neuroendocrine differentiation.	30962287
FOXC2	↗	EMT	FOXC2 is an important marker for aggressive PCa.	31464093
miR-221/222	N/A	AR	MiR-221/222 inhibits PCa cells migration and invasion and can be a biomarker for disease progression.	26325107
STAT3	↗	N/A	The over-expression of STAT3 is associated with CRPC bone metastases and poor prognosis.	27344294
MUC1	↗	N/A	High MUC1 expression is related to bone metastasis and castration resistance in PCa.	28930697
LY6D	N/A	N/A	LY6D a marker of castrate-resistant prostate progenitors, and its expression is associated with the progression of CRPC.	30566873
RGS2	↗	N/A	Elevated RGS2 is related to aggressive CRPC and suggests poor prognosis.	32449815
CPT1B	↗	AR	The expression of CPT1B is negatively correlated with the prognosis of patients, and it usually highly expresses in PCa patients.	32648618
miR-1290, miR-375	↗	N/A	Exosomal miR-1290/375 can be used as a prognostic biomarker for CRPC.	25129854
CLDN3	loss	N/A	CLDN3 can be used as a molecular marker for the prognosis of PCa patients and to distinguish aggressive from indolent PCa.	36614243

N/A, not applicable; ↗, high expression.

Bone metastasis has a negative effect on patient quality of life and contributes to fatal outcomes. Hence, timely intervention holds immense significance. As shown in **Table 3**, studies have shown that STAT3 and MUC1 were closely related to bone metastasis in CRPC patients (125, 126). The expressions of LY6D, RGS2, CPT1B, miR-1290, and miR-375 were related to the prognosis of CRPC patients and could be used as potential indicators for predicting prognosis (127–129). In addition, MED15 and stanniocalcin 2 were found to be overexpressed in CRPC and aggressive PCa, while they were expressed at lower levels in benign prostate tissue (130, 131). Furthermore, SLPI was a potential biomarker in the cell proliferation of CRPC under androgen deprivation conditions and its levels were observed to be increased significantly in mCRPC (132). SOX7 and SOX9 belong to the same SOX gene family, however, during castration resistance, SOX9 was found to be significantly increased, while SOX7 was observed to decrease significantly (133).

The identification of biomarkers holds both theoretical and clinical significance for CRPC risk prediction and personalized therapy. For example, the integration of biomarkers including HSD3B1, PHF8, Gal-1, and the SOX gene family facilitated the construction of computational models for CRPC early diagnosis (116, 119). The utilization of factors including LIF, NR6A1, miR-32, FOXC2, and miRNA-221/222 could improve the stratification of patients for applying personalized clinical therapeutics (122–124). Moreover, STAT3, and MUC1 indicated the possibility of bone metastasis, which would be helpful of monitoring the prognosis of CRPC patients into metastatic status (125, 126).

## Translational perspectives toward CRPC holistic healthcare

### Perspective 1: improving both AR and non-AR-targeted precision molecular therapy

**Figure 5:**
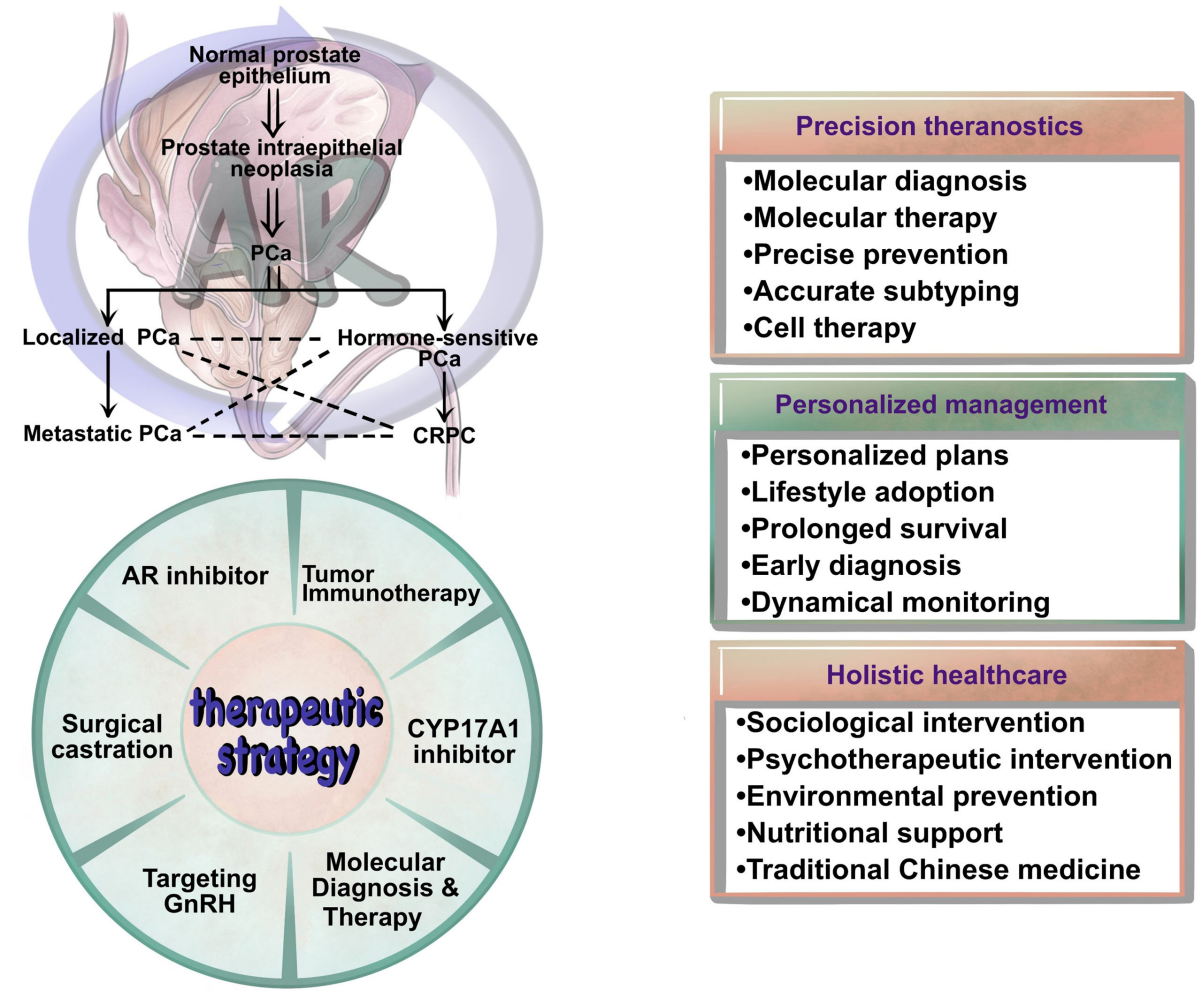
Translational perspectives for CRPC precision medicine and personalized therapy.

AR plays a pivotal role in PCa, particularly in cases of clinical CRPC. In PCa cell models, AR overexpression has been frequently observed and established as a primary driving factor for PCa progression (134). Over the past few decades, numerous anti-AR drugs have been developed and approved for use across different stages of PCa. In the 1980s and 1990s, the FDA approved the first-generation AR antagonists, including flutamide, nilutamide, and bicalutamide, which had efficacy in the early stages of the disease but ultimately led to the development of resistance and progression to CRPC. With the in-depth research into the AR, second-generation AR antagonists that target the ligand-binding domain (LBD), such as Enzalutamide, Apalutamide, and Darolutamide, have been developed and applied. These agents possess higher AR binding affinity, allowing for more effective suppression of AR expression, and have led to significant improvements in patient survival rates (135–137). However, with the rapid development of resistance, these drugs only provide short-term effects and may potentially give rise to central nervous system toxicity and cardiovascular toxicity (138, 139). It is acknowledged that PCa demonstrates significant inter- and intra-tumor heterogeneity. Targeting a single molecule (e.g., AR) does not benefit all patients, and does not affect all tumor cells equally. Recent studies indicated that many non-AR-based mechanisms were involved in CRPC development, including neuroendocrine cell-related mechanisms, prostate stem cell-related mechanisms, alternations in TME, and deregulations in non-AR genes and pathways. Understanding the role of non-AR-based mechanisms in the development of castration resistance in PCa is also important for identifying new therapeutic targets or strategies against castration resistance. Moreover, as shown in **Figure 5** the integration of both AR and non-AR strategies, e.g., inhibiting of AR and neuroendocrine cell-expressed CXCR2 simultaneously, may achieve a better therapeutic effect on CRPC (19).

### Perspective 2: identifying molecular mechanisms based on novel programmed cell death types for CRPC personalized medicine

Tumor cells demonstrate the ability to evade apoptosis, which is an important cause of drug resistance and recurrence in cancer therapy. In recent years, novel regulated cell death pathways such as ferroptosis and pyroptosis have gained increasing attention as representatives in cancer drug discovery and application (140). In PCa studies, Wang et al. triggered ferroptosis in mice using a stable GPX4 inhibitor in a genetically engineered model, and it inhibited the growth and spread of RB-deficient PCa tumors. This finding offered promising prospects for the treatment of RB1-deficient malignant PCa (141). Wu et al. confirmed that inhibiting CDC20 could promote pyroptosis in PCa cells and boost tumor immunity in a mouse model of PCa (142). Wang et al. synthesized a series of aggregation-induced emission materials to mediate the process of ferroptosis and pyroptosis for enhancing PCa immunotherapy (143, 144). As shown in **Figure 5**, the identification and application of novel programmed cell death-based approaches would be an emerging direction for CRPC treatment, especially for patients with failure under traditional CRPC therapeutics.

### Perspective 3: integrating multi-omics data and artificial intelligence for CRPC systems modeling and clinical application

Identifying molecular targets and understanding how these targets vary among different patient subgroups or racial groups and how this affects treatment outcomes are of clinical interest for CRPC personalized management. It is reported that there was a higher similarity at pathway level than that at single gene level in the expression of genes across different PCa datasets, which could partly explain why the single-gene based approaches cannot benefit all patient cohorts and indicate the significance for the development of network medicine-based strategies to fight against therapeutic heterogeneity in cancers (145). In the era of big data and artificial intelligence (AI), computer-aided modeling has now become an emerging approach for translational cancer researches. Compared with traditional experimental methods, computational algorithms simulate the diversity and dynamicity of disease occurrence and progression under a systems biology framework, which would promote the identification and characterization of key signatures for disease early diagnosis and personalized therapy (146, 147). The development of CRPC is a heterogenous process in which genetic, epigenetic, and environmental factors generate large-scale biological networks and contribute to the complexity in PCa phenotype from androgen dependence to castration resistance, thus it is of great significance to integrate multi-omics molecular data with image and clinical information as prior knowledge for multi-step AI model training (148). Here the AI models could be simply divided as two sub-categories based on the methods of feature selection, i.e., traditional models that manually characterize features for training, and deep learning-based techniques automatically extracting features for optimization. The typical applications of AI models for PCa studies are pathological evaluation and classification of multiple PCa status such as benign and malignant lesion identification, PCa grading and molecular subtyping, prognosis and risk stratification, prediction of time to CRPC (149–151), etc. Although there is a promising perspective of AI modeling in PCa and CRPC, the limitations and challenges are still worthy to be concerned. First, the clinical data have the characteristics of small sample size but high dimension and heterogeneity, hence how to reduce the overfitting results and address difficulties in model generalization are the leading issues to be considered. Second, the quality of datasets will directly affect the accuracy of model output. Currently, there is still a lack of in-depth research on clinical data standardization and privacy protection. Construction of PCa-related ontologies would be a possible and feasible way to provide a systematical framework for decoding the large amounts of PCa data and knowledge, and this will contribute to the development of data sharing and integration for model analyses (152). Finally, the interpretability of AI needs to be improved continually, and clinical urologists and pathologists should strengthen their professional behaviors to avoid the biases of missed diagnosis caused by AI models (153).

## Conclusions

Although there has been a notable advancement in the field of CRPC research, the current clinical management of CRPC remains a challenge. The emergence of CRPC tumors is predominantly propelled by genetic and molecular events. For instance, accumulating evidence confirmed the role of AR signaling in the progression of PCa to castration resistance. However, the evolution of CRPC is a complex and dynamic process, and AR signaling is not the only clue for CRPC understanding. Hence, it is urgently needed for further elucidating the pathogenesis of CRPC by integrating molecular signatures at muti-omics levels. This review provides an updated landscape of literature-reported molecules for CRPC, which may offer novel insights and targets for translational CRPC research to facilitate the early diagnosis and personalized therapeutics of CRPC.

## Author contributions

JJ: Writing – review & editing, Writing – original draft, Validation, Formal analysis, Data curation. XW: Writing – review & editing, Writing – original draft, Validation, Formal analysis, Data curation. JZ: Writing – original draft, Formal analysis, Data curation. CZ: Writing – original draft, Formal analysis. XH: Writing – original draft, Formal analysis. YH: Writing – review & editing, Validation, Funding acquisition. JH: Writing – review & editing, Validation, Supervision, Conceptualization. YL: Writing – review & editing, Writing – original draft, Validation, Supervision, Funding acquisition, Conceptualization. XW: Writing – review & editing, Writing – original draft, Validation, Supervision, Conceptualization.
